# Moyamoya Disease Mimicking Encephalitis

**Published:** 2014-09

**Authors:** Maryam Khalesi, Masoud Pezeshki Rad, Abdolkarim Hamedi, Mohammad Hassan Aelami

**Affiliations:** 1 Department of Pediatrics, Ghaem Medical Center, Mashhad University of Medical Sciences, Mashhad, Iran;; 2 Department of Radiology, Imam Reza hospital, Mashhad University of Medical Sciences, Mashhad, Iran;; 3 Department of Pediatrics, Imam Reza Hospital, Mashhad University of Medical Sciences, Mashhad, Iran

**Keywords:** Moyamoya disease, Child, Encephalitis

## Abstract

Moyamoya disease is a rare vaso-occlusive illness with an unknown etiology characterized by stenosis of the internal carotid arteries with spontaneous development of a collateral vascular network.

A 15-month-old girl was referred to the emergency ward of Imam Reza Hospital due to decreased level of consciousness, focal seizures and fever during the previous 24 hours with an impression of encephalitis. Physical examination revealed left side hemiparesis; however brain CT-Scan did not show any significant lesions. Initial therapy with vancomycin, ceftriaxone and acyclovir was administered. CSF analysis did not show any abnormality and the blood as well as CSF cultures results were negative. Brain MRI showed hyperintensity at right frontal and parietal regions, suggesting vascular lesion. Magnetic resonance angiography (MRA) showed bilaterally multiple torsions in vessels at the basal ganglia consistent with moyamoya vessels.

In all children exhibiting encephalitis, vascular events such as moyamoya disease should be considered. Brain MRI is a critical tool for this purpose. Common causes of encephalitis such as herpes simplex should also be ruled out.

## Introduction

Moyamoya disease is a rare occlusive disorder of the cerebral vasculature. “Moyamoya” is a Japanese word meaning puffy, obscure, or hazy like a puff of smoke in the air. The term describes smoky appearance of the vascular collateral network. There is a progressive stenosis of terminal parts of bilateral internal carotid arteries and the main trunks of anterior and middle cerebral arteries, resulting in the formation of collateral vessels at the base of the brain.

There are various clinical manifestations for moyamoya disease. Two major categories of symptoms are described as; those due to brain ischemia (i.e. stroke, transient ischemic attacks and seizures) and those due to the deleterious consequences of compensatory mechanisms responding to the ischemia (i.e. hemorrhage and headache from dilated transdural collaterals).^1^

Moyamoya cases are mainly reported from Japan (prevalence of 3 cases per 100,000 children). This disease occurs less frequently in North America and Europe an countries compared with the Asian countries. The incidence of moyamoya disease in Europe appears to be about 1/10th of those observed in Japan.^2^

This case report describes a child with moyamoya disease which was admitted with the impression of encephalitis, being not a common manifestation of moyamoya disease.

## Case Report

**Figure 1:**
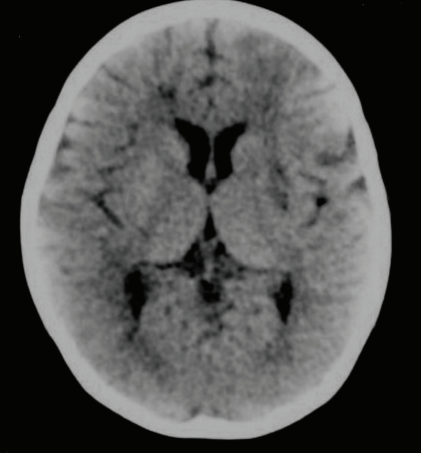
Axial brain CT scan showing no significant lesion.

**Figure 2:**
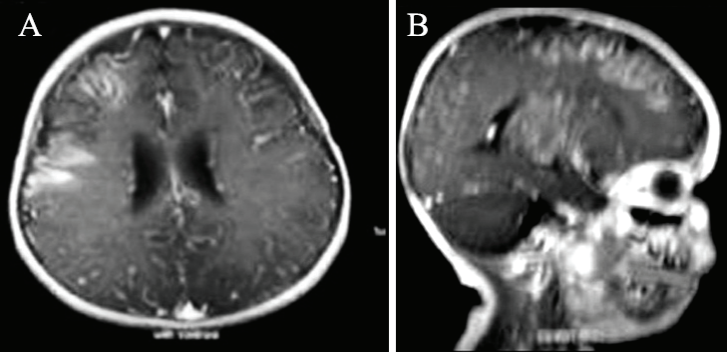
Axial (a) and coronal (b) post contrast T1-weighted images showing diffuse leptomeningeal enhancement (ivy sign) at the right frontoparietal region.

**Figure 3:**
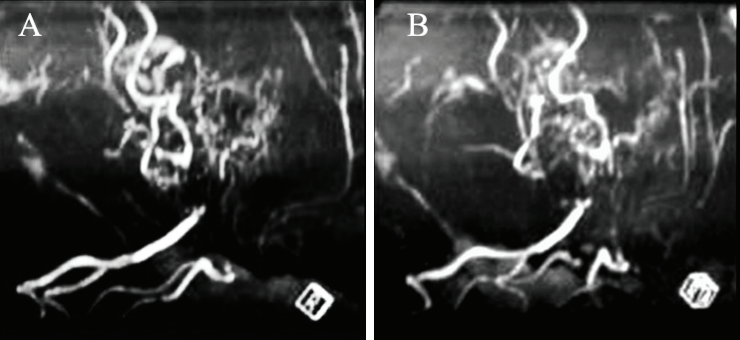
Magnetic resonance angiography images showing replacement of normal intracranial arteries with multiple moyamoya vessels.

A 15-month-old girl was referred to the emergency ward of Imam Reza Hospital (Mashhad-Iran) in May 2011, due to decreased level of consciousness, focal seizures and fever. During the previous 24 hours, she was in good health after which she developed symptoms of common cold (fever, cough and rhinorrhea). She is the third child of non-consanguineous parents. Additionally, when her elder sister was three years old, she was also admitted to hospital with similar symptoms of fever and hemiparesis without any specific diagnosis. The patient underwent lumbar puncture and was treated with systemic antibiotics and acyclovir. On the second day, there was improvement on consciousness status but physical examinations revealed left side hemiparesis. Axial brain CT-Scan did not show any significant lesion (figure 1). CSF evaluation including analysis, bacterial culture and PCR of herpes simplex virus as well as blood culture were all negative. Brain MRI showed linear high signals that follow a sulcal patterns due to leptomeningeal enhancement in post contrast sequences (figure 2). Laboratory work up for stroke including ANA, protein C, factor V Leiden, antiphospholipid antibody, antithrombin III, triglyceride, cholesterol, PT, PTT and hemoglobin electrophoresis were normal. EEG showed epileptiform activity. Magnetic resonance angiography (MRA) showed reduced flow voids in the internal, middle and anterior cerebral arteries and bilaterally multiple torsions in vessels at the basal ganglia (moyamoya like vessels) consistent with moyamoya disease (figure 3). Diagnosis of moyamoya disease was confirmed based on MRA findings and family history (similar attack with her sister). The patient was managed conservatively and referred to neurosurgery ward.

## Discussion

Moyamoya disease was reported in Japan for the first time in 1957. Decreased blood flow in the main vessels of the brain would result in the formation of collateral vasculature at the apex of the carotid, on the cortical surface, leptomeninges and branches of the external carotid artery.^1^

The patient under discussion exhibited fever, seizure and decreased level of consciousness that was misdiagnosed with viral encephalitis especially HSV. Common clinical manifestations of moyamoya are reported to vary and include transient ischemic attack (TIA), ischemic stroke, hemorrhagic stroke and epilepsy. The expression of the disease might be different depending on the age at the time of diagnosis. Ischemic cerebrovascular events, either infarction or TIA, are more prevalent than hemorrhagic events in children.^1^ In some studies the most common symptoms at presentation are ischemic stroke (50-75%), transient ischemic attack (50-75%) and hemorrhagic attack (10-40%). Children have high rate of complete stroke than TIA, which might be due to their immature verbal and reporting skills. As they may not be able to communicate TIA symptoms clearly, it may delay diagnosis and increase the likelihood of complete stroke.^2^ Shoukat et al. reported a case series of patients with moyamoya disease where 6 out of 13 patients presented by fever and seizure after which received lumbar puncture to rule out encephalitis. Fever is a common symptom in the lower age group (n=4, 51.7%).^3^ The above mentioned symptoms are similar to the patient in this report but without decreased level of consciousness. Suyama et al. reported three cases of moyamoya disease presented with headache and vomiting, in which one patient became lethargic during hospitalization.^4^ This presentation could be a manifestation of encephalitis similar to the patient in this case study. Therefore, it is essential to consider moyamoya disease as a differential diagnosis of encephalitis.

Moyamoya disease is reported to be primarily present at an early age (typically less than 10 years old) and is more common among females than males (M/F ratio of 1/1.8). This statement is in-line with the patient in this report and her sibling as they also exhibited the disease during their childhood. The second peak of moyamoya disease during the fourth decade has also been reported in the literature.^2^ Mean age is reported in Shokat study to be at 16.5 and in Suyama study at 7.6 years old.^3^^,^^4^

The high occurrences of moyamoya disease in Japan and Asian countries, along with positive familial history in about 10% of cases, support a genetic background.^5^ Moyamoya disease is rarely reported amongst the Iranian population, which can probably be due to under diagnosis. This argument is supported by the fact that the sibling of the patient in this study was not diagnosed for her potential moyamoya disease. Diagnosis requires expressive suspicion and radiological studies.

Considering the family history of this patient (i.e. disease in affected sibling despite lack of any symptoms in her parents), there is indication of familial form of moyamoya disease. In a study carried out by Nanba et al. family screening of 141 moyamoya patients showed 14 new cases.^6^ Familial moyamoya disease is related to chromosomes 3p24.2-p26, 8q23, 6q25, 12p12, and 17q25. Familial moyamoya is reported as an autosomal dominant disease with incomplete penetrance.^5^ For the case of the patient in this investigation, genetic evaluation was not carried out.

Traditionally, patients with the angiographic appearance of moyamoya and no known risk factors are considered to have moyamoya disease, while those with one of the well-recognized associated conditions (neurofibromatosis type 1, cranial irradiation, Down’s syndrome, and sickle cell disease) are classified as having moyamoya syndrome. This syndrome may also be the result of infectious diseases.^7^

To the best knowledge of the authors, widespread screening for moyamoya is not yet standard for any specific group. However, diagnosis should be considered when a family member is affected or in patients with certain high-risk disorders as mentioned above.

Initial axial brain CT scan for the patient in this investigation was normal, which is commonly observed in early stages of ischemic attacks and encephalitis. Axial brain CT scan typically shows an area of hyperdensity or hypodensity due to hemorrhagic or ischemic events in other study.^4^

MRI findings showed a pattern of increased signal in the right frontotemporal region and increased signal in the leptomeningeal and perivascular space in FLAIR sequences. This pattern has been termed as “ivy sign”, since it resembles the appearance of ivy creeping on stones. The probable cause is slow retrograde collateral flow through engorged pial vessels via leptomeningeal anastomosis. The ivy sign is correlated with decreased cerebrovascular reserve.^8^ Similar findings are also reported in other studies.^1^^,^^3^^,^^4^^,^^6^

MRA is a useful and noninvasive diagnostic method showing stenotic and collateral vessels at the base of the brain.^5^ In this case report, MRA showed typical moyamoya associated collateral vessels through the basal ganglia and thalamus. In a study by Shokat et al. MRI and MRA was carried out on 13 cases, 11 (84.6%) had bilateral stenotic vessels on MRA or cerebral angiograms.^3^

Three EEG patterns are reported in moyamoya disease.^1^^,^^2^ The patient in this case report had evidence of epileptiform activity probably due to focal ischemia. Shoukat et al. performed EEG on 6 patients (46.1%) with moyamoa disease. Four patients (30.8%) showed diffuse cerebral dysfunction and two (15.4%) had an epileptiform activity, with one being focal and the other non-focal.^3^

Irrespective of radiological methods used for diagnosis, conditions like von reckling hausen’s disease, down’s syndrome, autoimmune vasculitis, head trauma, meningitis, history of cranial radiation and brain tumor may have a similar picture.^2^ The patient in this case report did not exhibit any of the above conditions.

## Conclusion

Moyamoya disease should be considered in a child with fever, decreased level of consciousness and/or seizures suggestive of encephalitis especially when CSF analysis and other laboratory tests does not confirm the diagnosis. 
